# MiR-130c-5p targets the SHVV *n* gene and upregulates immune cytokines (IL-6, IL-22, IL-1β) to inhibit viral replication

**DOI:** 10.3389/fimmu.2024.1486816

**Published:** 2024-11-01

**Authors:** Jin Wei, Yan Ji, Yaqian Bai, Rui Cheng, Jiaqi Zhang, Xianqin Hu, Chi Zhang

**Affiliations:** ^1^ Hubei Key Laboratory of Animal Nutrition and Feed Science, School of Animal Science and Nutritional Engineering, Wuhan Polytechnic University, Wuhan, China; ^2^ Key Laboratory of Ecological Impacts of Hydraulic-Projects and Restoration of Aquatic Ecosystem of Ministry of Water Resources, Institute of Hydroecology, Ministry of Water Resources and Chinese Academy of Sciences, Wuhan, China

**Keywords:** miR-130c-5p, *Snakehead fish Vesiculovirus*, targeting, nucleoprotein, antiviral immunity

## Abstract

**Background:**

*Snakehead vesiculovirus* (SHVV) has led to huge economic losses in snakehead aquaculture, and its pathogenic mechanisms is still not fully understood. MicroRNAs (miRNAs), as an important class of non-coding RNAs, play a key regulatory role in the process of viral infection.

**Methods:**

We examined the effect of SHVV infection on the expression of miR-130c-5p and the effect of overexpression of miR-130c-5p on the proliferation of SHVV. Cotransfection of viral N protein and miR-130c-5p, and the effect of miR-130c-5p on the expression of N protein was detected. Meanwhile, the effect of overexpression of miR-130c-5p on the expression of various immune factors in the case of viral infection were also tested.

**Results:**

In this study, SHVV infection significantly upregulated the expression of miR-130c-5p in channel catfish ovary (CCO) cells in a time- and dose-dependent manner. The further research revealed that miR-130c-5p mimic significantly inhibited, while its inhibitors promoted SHVV replication. In addition, miR-130c-5p could directly target the viral mRNA of *n* gene, and overexpression of miR-130c-5p could significantly decrease, and conversely, downregulation of miR-130c-5p could increase the mRNA and protein expression of the viral n gene. Meanwhile, overexpression of miR-130c-5p also upregulated the expression of immune-related genes, such as nucleotide-oligomerization domain (NOD)-like receptor subfamily C3 (NLRC3), myeloid differentiation factor 88 (MyD88), nuclear factor kappa-B (NF-κB), interleukin-6 (IL-6), interleukin-22 (IL-22), and interleukin-1beta (IL-1β) in host cells.

**Conclusion:**

miR-130c-5p was upregulated in the host during SHVV infection, and the upregulated miR-130c-5p directly inhibited viral replication by targeting the *n* gene of SHVV and promoting viral nucleoprotein degradation. The up-regulated miR-130c-5p also activated the expression of immune-related genes such as NLRC3, MyD88, NF-κB, IL-6, IL-22, and IL-1β, which were involved in the regulation of the signaling pathways including NF-κB, MyD88, Toll-like receptor (TLR), NLR, and janus tyrosine kinase-signal converter and activator of transcription (JAK-STAT), to enhance the host's antiviral immune response, and thus indirectly inhibited the viral proliferation.

## 1 Introduction

*Snakehead fish vesiculovirus* (SHVV) is a member of the *Rhabdoviridae*, which are important viral pathogens causing lethal and epidemic diseases in fish and other aquatic animals, and its genome is a single-stranded, unsegmented, negative-stranded RNA of about 11 kb (1, 2). The virus encodes five main structural proteins: nucleoprotein (N), phosphoprotein (P), matrix protein (M), glycoprotein (G), and RNA-dependent RNA polymerase protein (L) (3). Among them, nucleoprotein not only serves as a template for viral transcription and replication, but also binds to viral genomic RNA, participates in the assembly process of viral particles, and plays an important role in regulating the transcription and replication process (4, 5). It was demonstrated that the interaction of the N protein of the SHVV virus with its leading RNA produced during infection promotes viral replication (6). After the virus has invaded the fish, the nucleoprotein will be recognized by the immune system as an antigen, activating T lymphocytes, especially T helper lymphocytes, and promoting the production of specific antibodies against the virus by B lymphocytes (7). In addition, the lethality of the disease is extremely high, up to 90% or more, but there is a lack of effective control and treatment measures (8, 9).

During viral infection, a series of changes in the expression profile of cellular miRNAs are triggered, and these differentially expressed miRNAs play an important regulatory role in viral invasion as well as host immune defense mechanisms. MiRNAs, as a class of highly conserved, non-coding small RNA molecules, mainly rely on the 2nd-8th nucleotides at the 5’ end (seed sequence) to recognize the mRNA of the target gene and bind it by base complementary pairing to degrade or inhibit the translation of the target gene (10, 11). Numerous studies have demonstrated that miRNAs are involved in regulating viral replication in a direct or indirect manner during viral infection. For example, gallus gallus miRNAs (gga-miR-1603 and gga-miR-1794) directly bind to the *l* gene of *Newcastle disease virus* to facilitate ts degradation and inhibit the replication of multiple genotypes of *Newcastle disease virus* (NDVs) *in vitro* (12); miR-370 inhibits *Hepatitis B virus* (HBV) gene expression and replication by targeting transcription factor nuclear factor IA (NFIA), whereas NFIA stimulates HBV replication by directly regulating the activity of HBV enhancer I (13); *Recombinant Snakehead Rhabdovirus* (SHRV) can express artificial microRNAs targeting *Spring Viremia of Carp Virus* (SVCV) p gene to treat SVCV infection (14). Furthermore, miRNAs can indirectly affect the host’s antiviral immune response by regulating intracellular signaling pathways, such as interleukin (IL)-mediated signaling pathways (15, 16) and interferon (IFN)-mediated signaling pathways (17, 18).

Recently, SHVV infection has led to hundreds of millions of dollars of annual economic losses in cultured snakeheads, but its pathogenic mechanism and interaction with host cells have not been fully elucidated (19). Based on the previous study that channel catfish ovary (CCO) cells are experimental materials with high susceptibility to SHVV (20), we screened the microRNA expression profiles of host cells by sequencing and found that SHVV infection alters the expression of host miR-130c-5p, but its role in SHVV infection is yet to be explored in depth (21). In this study, we explored the effect of miR-130c-5p on viral proliferation and host immune response after SHVV infection with CCO to deepen our understanding of the molecular mechanism of SHVV infection.

## 2 Methods

### 2.1 Cells and viruses

CCO cells were cultured in minimum essential medium (MEM) (Gibco, New Zealand) containing 10% fetal bovine serum (FBS) (Yeasen Biotechnology, Shanghai) and 1% penicillin-streptomycin mixture (PBS) (Life ilab Biotechnology, Shanghai) in medium at 25°C in a constant temperature incubator (Gibco, New Zealand) in medium at 25°C in a constant temperature incubator. SHVV was isolated from diseased hybrid snakeheads collected from a fishery in Guangdong Province, China, and stored at -80°C.

### 2.2 Reagents and antibodies

The miR-130c-5p mimic (double-stranded RNA oligonucleotides), miR-130c-5p inhibitor (single-stranded chemically modified oligonucleotides), negative control (NC) mimic, and NC inhibitor were purchased from GenePharma (Shanghai, China). Their sequences were as follows: miR-130c-5p mimic, 5’- UAGCUUAUCAGACUGAUGUUGA-3’ (forward) and 5’-AACAUCAGUCUGAUAAGCUAUU-3’ (reverse); miR-130c-5p inhibitor, 5’- UCAACAUCAGUCUGAUAAGCUA-3’; NC mimic, 5’- UUCUCCGAACGUGUCACGUTT-3’ (forward) and 5’-ACGUGACACGUUCGGAGAATT-3’ (reverse); NC inhibitor, 5’-CAGUACUUUUGUGUAGUACAA-3’.

Antibodies against SHVV N have been prepared and stored in our laboratory. In the experiment, the following antibodies were used: *β-actin* antibody, primary anti-rabbit GFP and secondary anti-rabbit HRP antibodies), all of which were purchased from Proteintech Group (Wuhan, China).

### 2.3 Plasmids

The plasmid pCDNA3.1-N expressing SHVV N was constructed by cloning the pcr-amplified cDNA of N into the vector pCDNA3.1(+) using the primers shown in **Table 1**. The 130c-5p target sequence (~200 nt) of the coding region of the *n* gene (GenBank: OQ211330.1) was amplified and cloned into the pmirGLO vector using the primers shown in **Table 1** to construct the luciferase reporter plasmid pmirGLO-N. Plasmid pmirGLO-N-MUT was generated by PCR-mediated mutation of plasmid pmirGLO-N using the primers listed in **Table 1**.

**Table 1:** Primers used in this study.

Primer*	Sequences (5’→3’)
SHVV-N-FW	CCGCATCGGAAATCAAGCAG
SHVV-N-BW	GTTGACCGCTTGCCCAATTT
NLRC3-FW	TGGCTTCCAAAACCACTATCG
NLRC3-RW	ACCGCCTCGCCTCCTGAT
MyD88-FW	AAGAGGATGGTGGTCGTCA
MyD88-BW	AGGAATCAGCCGTTTGGT
NF-Kb-FW	CCTCATCAATGCCTTCCG
NF-Kb-BW	CGCTGTTCCCGATACTCTT
IL-6-FW	CAGCCCGCAAAAATGTCTGC
IL-6-RW	TCAGGTAAGGAGGTCGGGCG
IL-22-FW	GCGCTGTACTTGCTGTGCTG
IL-22-RW	CGCTTGCGCGTGCTTGGCCA
IL-1β-FW	TGAGAATGTGATTGAAGAGAGC
IL-1β-BW	TTGTTTCCACCCTTCAGAGT
β-actin-FW	GCCCATCTCCTGCTCAAAG
β-actin-BW	CCATCTCCTGCTCGAAGTC
miR-130c-5p-FW	GCCCTTTTTCTGTTGTACTACT
U6-FW	CTCGCTTCGGCAGCACA
U6-BW	AACGCTTCACGAATTTGCGT
N-FW	CCGCTCGAGATCGTGGTCCGCTATCTGCT
N-BW	GCGTCGACGCACTCCACTGAGGGTT
N-MUT-FW	TAGGATCTGCCTCTAGGGGGACGACCTGGATTGCATCCATGAC
N-MUT-BW	GTCGTCC CCCTAGAGGCAGATCCTA

*Primers with names beginning with N- were used to generate luciferase reporter plasmids. Other primers were detected by qRT-PCR.

### 2.4 Transfection

The NC mimic, NC inhibitor, miR-130c-5p mimic, miR-130c-5p inhibitor, and pCDNA3.1-N were incubated with PEI Transfection Reagent (Yeasen Biotechnology, Shanghai) in 50 µL Opti-MEM medium (Yeasen Biotechnology, Shanghai) for 30 minutes at room temperature. The incubated samples were then inoculated onto CCO cells. After 4-6 hours of transfection at 25°C, replace with new cell culture medium. Cell samples are collected at the required time for subsequent experiments.

### 2.5 Dual-luciferase reporter assay

CCO cells were co-transfected with 30 pmol of NC mimic, miR-130c-5p mimic, NC inhibitor or miR-130c-5p inhibitor with 500 ng of luciferase reporter plasmids pmirGLO-N and pmirGLO-N mut using TransIntroTM EL transfection reagent (TransGen Biotech, China). co-transfected CCO cells. Twenty-four hours after transfection, luciferase activity was measured using the Dual-Glo luciferase assay system (Promega, USA). Measurements were performed using a GloMax-Multi - Jr single-tube multimode reader (Promega, USA) according to the manufacturer’s protocol. Data are expressed as relative firefly luciferase activity normalized to Renilla luciferase activity.

### 2.6 Virus infection and titration

CCO cells were incubated with SHVV at an MOI of 1. After adsorption for 2 h at 25°C, the inoculum was removed and the cells were washed twice with PBS, followed by the addition of MEM containing 5% FBS. Supernatants were collected at different time points for titration of virus with TCID50, and cells were harvested for detection of viral proteins by Western blot or for detection of relative viral mRNA expression by quantitative real-time PCR (qRT-PCR).

### 2.7 Quantitative real-time PCR

Total RNA from cell samples was extracted according to the instructions of the Extraction Reagent (Vazyme, Nanjin). RNA was reverse transcribed to obtain cDNA according to the instructions of the HiScript III RT SuperMix for qPCR (+gDNA wiper) Reverse Transcription Kit (Vazyme, Nanjin). *β-actin* was used as an internal reference gene for cellular and viral genes, and expression differences between different samples were calculated by the 2 ^-ΔΔCT^ method, and all data were expressed as relative expression of mRNA.

### 2.8 Western blotting

Proteins were extracted from CCO cells with cell lysis buffer, separated by SDS-PAGE gel and then transferred to nitrocellulose membranes (Biosharp, China). Membranes were blocked with 5% skimmed milk in tris-buffered saline (TBST) containing Tween 20 overnight at 4°C and then incubated with primary antibodies against *β-actin* (1:10 00) or SHVV protein (1:10 00) for 2 h at room temperature. After three washes with TBST, the cells were incubated with HRP-conjugated Affinipure goat anti-rabbit (1:10 000) for 1 h at room temperature. The signal intensity was then measured using a BeyoECL Star (Beyotime, Shanghai).

### 2.9 Statistical analysis

All statistical analyses were performed using GraphPad Prism 5.0 (GraphPad Software, CA, USA). The statistical significance of the data was determined by Student’s t-test, and P<0.05 was considered statistically significant.

## 3 Results

### 3.1 Effect of SHVV infection of CCO cells on N and miR-130c-5p expression

**Figure 1:**
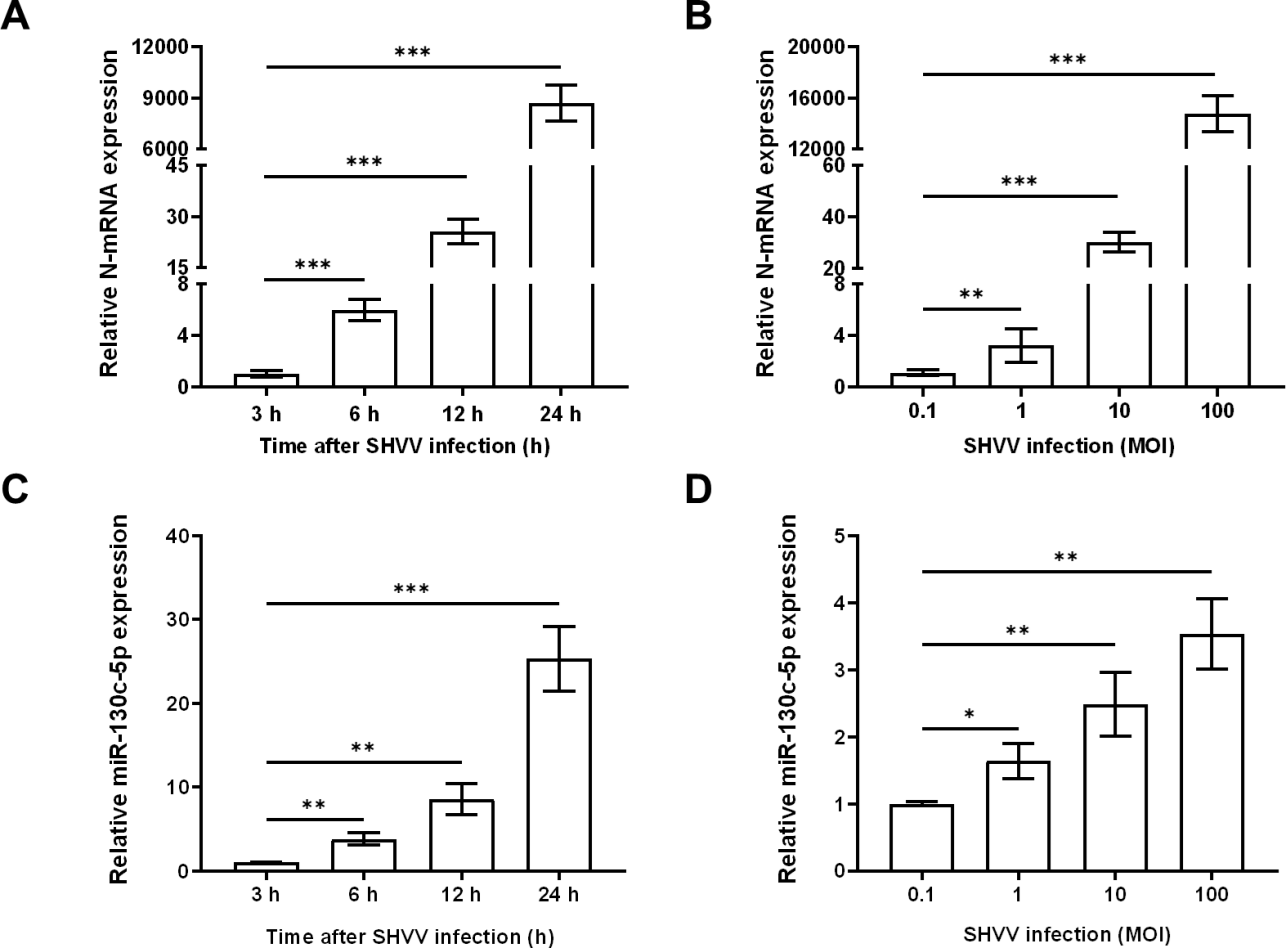
SHVV infection upregulated miR-130c-5p expression. **(A)** CCO cells were infected with SHVV (MOI=1) and harvested at 3 h, 6 h, 12 h, and 24 h. The expression level of viral n gene was detected using qRT-PCR and *β-actin* was used as an internal reference; **(B)** CCO cells were infected with SHVV (MOI=0. 1, 1, 10 and 100), and cells were harvested 24 h later using qRT-PCR to detect the expression level of the viral n gene in the cells and the *β-actin* was used as an internal reference. **(C)** CCO cells were infected with SHVV (MOI=1) for 3 h, 6 h, 12 h and 24 h later. The expression level of miR-130c-5p in cells was used by qRT-PCR, and the U6 was used as an internal reference; **(D)** CCO cells were infected with SHVV (MOI=0.1, 1, 10, 100) and harvested after 24 h. The expression level of miR-130c-5p in cells was measured using qRT-PCR, and the U6 was used as an internal reference. All data represent the results of at least two independent experiments, and each assay was performed in triplicate (mean ± SD); the same below (*, *p* < 0.05; **, *p* < 0.01; ***, *p* < 0.001).

A previous study of SHVV-infected miRNAs showed that miR-130c-5p was significantly altered in virus-infected host cells, and therefore, it was selected for the study. In addition, during the study of the SHVV reverse genetic system, CCO cells were found to support efficient SHVV replication. In order to investigate the role of miR-130c-5p in SHVV infection, we chose different infection times (3h, 6h, 12h, 24h) and different infection doses (MOI=0.1, 1, 10, 100) of SHVV to attack the CCO cells, and used qRT-PCR to measure the mRNA expression level of viral N protein. The results showed that the N protein mRNA of SHVV increased significantly at different infection times and doses (**Figures 1A, B**), indicating that SHVV could indeed replicate effectively in CCO cells. In addition, the level of miR-130c-5p was detected in SHVV infected CCO cells at different infection times and doses. The results showed that miR-130c-5p expression was significantly up-regulated in CCO cells relative to controls from 0 h to 24 h after viral infection; it was significantly up-regulated with the increase of viral infection dose (**Figures 1C, D**). These data suggest that SHVV infection caused upregulation of miR-130c-5p expression in CCO cells under certain conditions.

### 3.2 MiR-130c-5p inhibits SHVV replication

**Figure 2:**
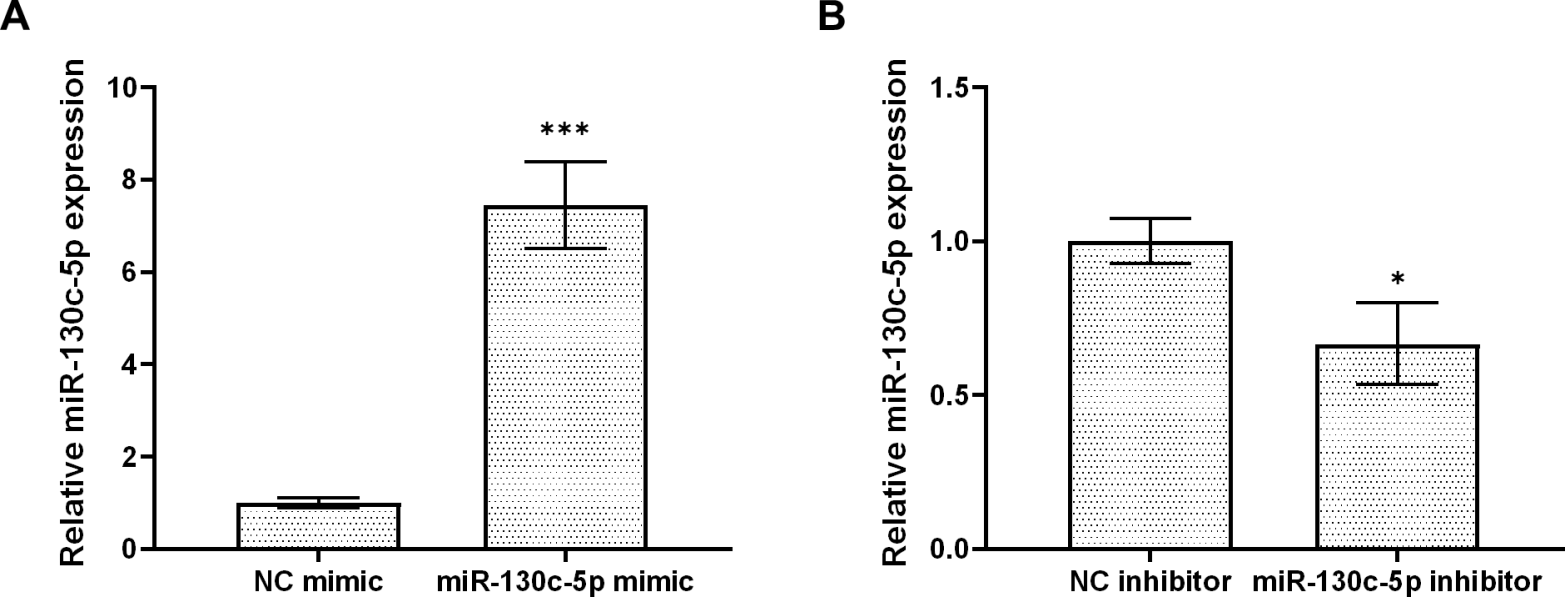
MiR-130c-5p inhibits SHVV replication. CCO cells were transfected with NC mimic, miR-130c-5p mimic, NC inhibitor, or miR-130c-5p inhibitor for 24 h, and the miR-130c-5p expression in CCO cells was measured using qRT-PCR. **(A)** miR-130c-5p analogue and its control; **(B)** miR-130c-5p inhibitor and its control. The U6 was used as an internal control, and all data are the results of at least two independent experiments, and each assay was performed in triplicate (mean ± SD); the same below (*, *p* < 0.05; ***, *p* < 0.001).

To determine whether miR-130c-5p is associated with SHVV replication, we transfected CCO cells with synthetic analogs of miR-130c-5p, inhibitors, or the corresponding negative control (NC), respectively, to overexpress or inhibit cytosolic miR-130c-5p, which was subsequently infected by SHVV 24 h post-transfection using qRT-PCR to detect the expression level of intracellular miR-130c-5p. Transfection with miR-130c-5p analogs resulted in a highly significant increase in intracellular miR-130c-5p expression levels compared to NC analogs (**Figure 2A**). Transfection of miR-130c-5p inhibitor resulted in a significant down-regulation of intracellular miR-130c-5p expression level compared to NC inhibitor (**Figure 2B**).

### 3.3 N of SHVV is a target gene of miR-130c-5p

**Figure 3:**
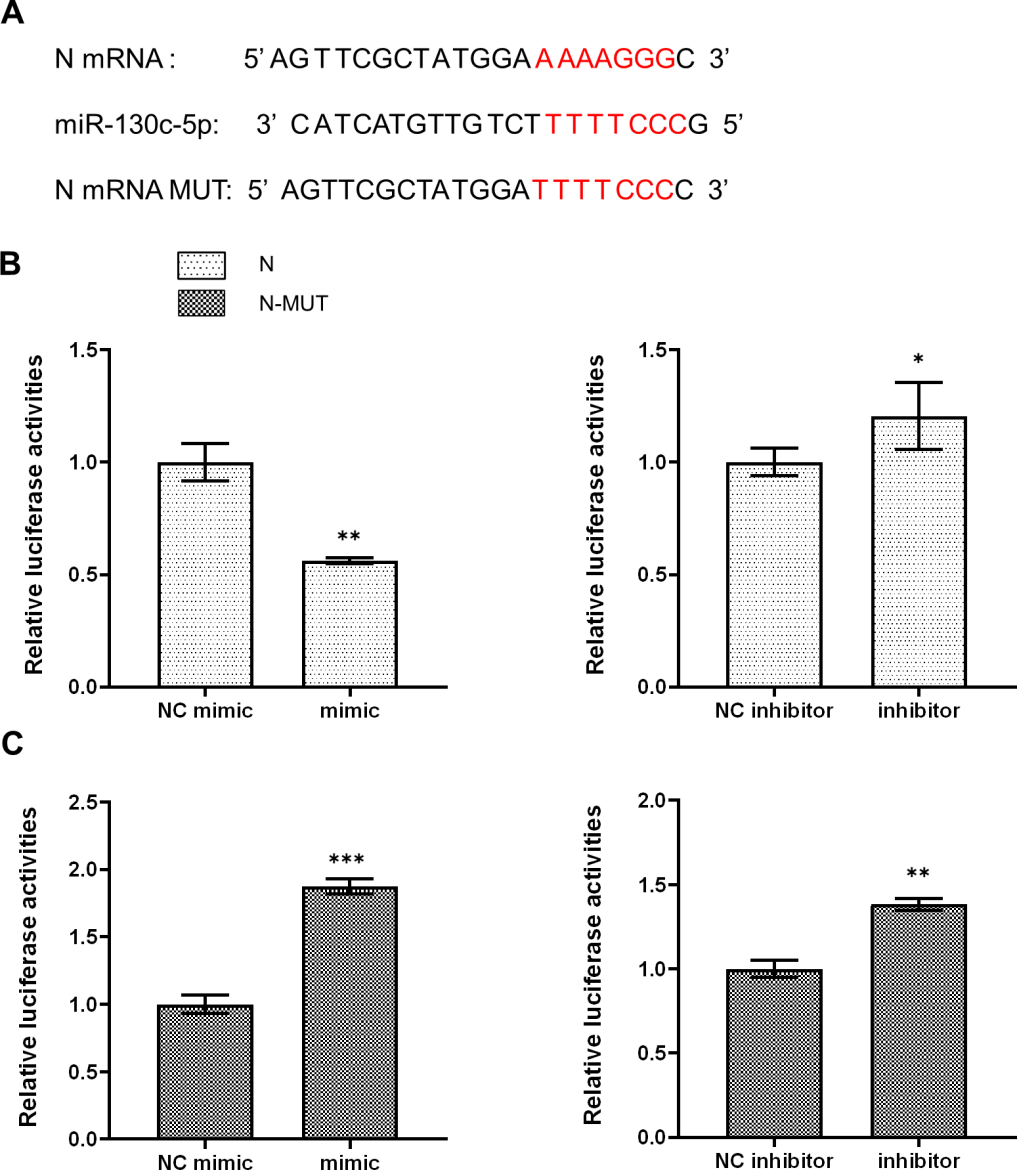
MiR-130c-5p targets the *n* gene of SHVV. **(A)** Base complementary pairing diagram of the predicted targeting sequences in the coding region of *n* mRNA; **(B, C)** CCO cells were co-transfected with miR-130c-5p mimic, miR-130c-5p inhibitor and its control with pmirGLO-N plasmid or PMIRGLO-N-MUT plasmid. The fluorescence intensity of the cells was measured 24 hours after transfection. All the data are representative of at least two independent experiments, with each determination performed in triplicate (mean ± SD); the same below (*, *p* < 0.05; **, *p* < 0.01; ***, *p* < 0.001).

Based on the “seed sequence” paired sequence approach to find target genes, we searched for and predicted the miR-130c-5p target gene from the full gene sequence of SHVV (NCBI NO. KP876483) in NCBI (**Figure 3A**). To verify whether the *n* gene was indeed a miR-130c-5p target gene, we performed dual luciferase reporter gene analysis. We transfected CCO cells with the dual-luciferase reporter plasmids pmirGLO-N and pmirGLO-N-MUT and transfected CCO cells with an analogue of miR-130c-5p, an inhibitor of miR-130c-5p, and its control. At 24 h post-transfection, the binding of miR-130c-5p to n-gene mRNA target sequences was verified by the dual luciferase reporter system. The results showed that the luciferase activity detected after transfection of miR-130c-5p mimic reduced, whereas miR-130c-5p inhibitor increased the luciferase activity (**Figures 3B, C**). However, there was no significant change in luciferase activity when miR-130c-5p mimic or inhibitor was cotransfected with plasmids containing mutant sequences in the seed region of miR-130c-5p (**Figures 3B, C**). These data suggest that the *n* gene of SHVV is indeed a target gene of miR-130c-5p and that the N protein region encodes a target sequence with miR-130c-5p.

### 3.4 MiR-130c-5p reduces mRNA and protein expression levels of SHVV N

**Figure 4:**
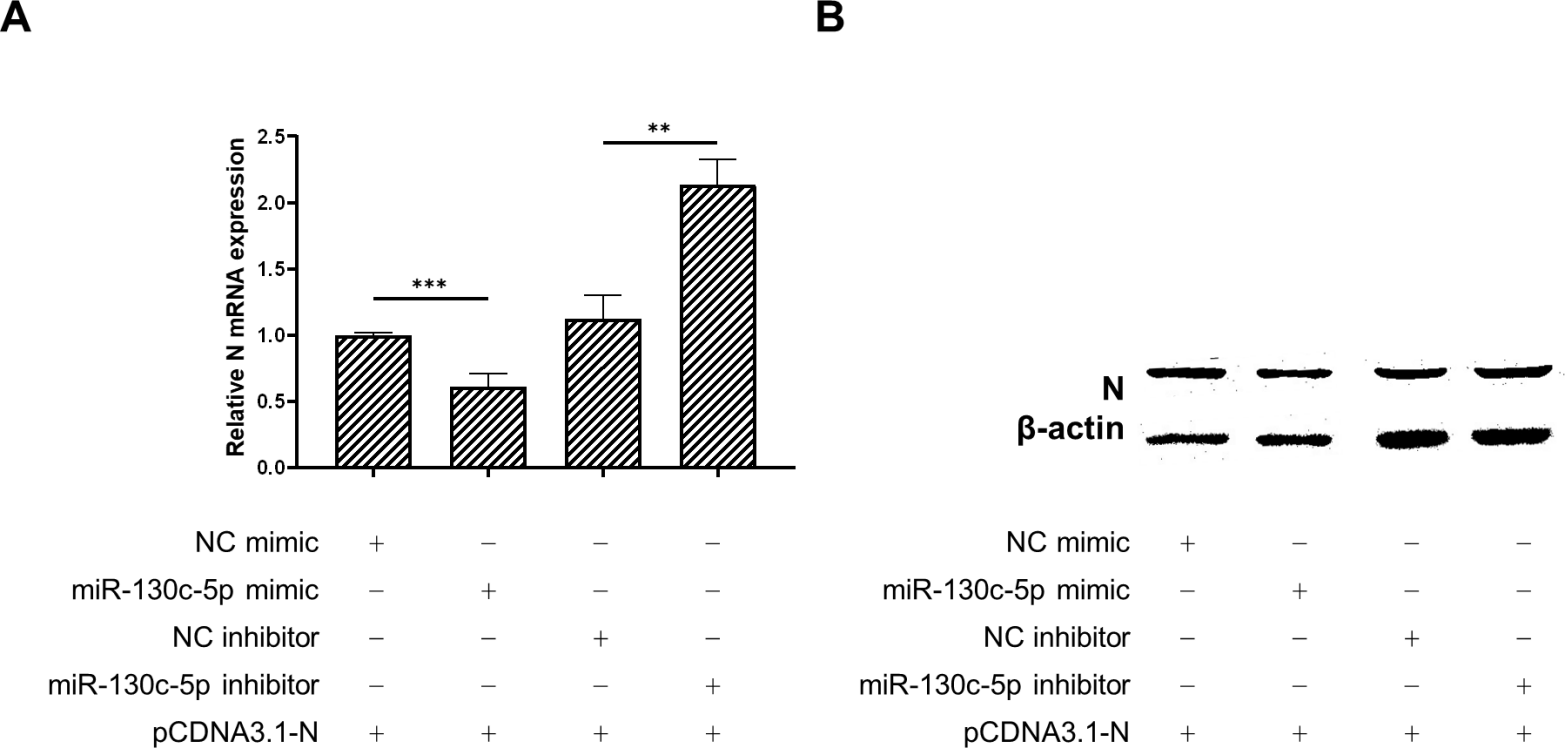
MiR-130c-5p reduced the mRNA and protein expression of SHVV N. CCO cells were co-transfected with analogues of miR-130c-5p, inhibitors and their controls and the constructed plasmid pCDNA3.1-N for 24 h. **(A)** The expression level of *n* mRNA was detected in CCO cells using qRT-PCR, with the *β-actin* as an internal reference. All the data are representative of at least two independent experiments, with each determination performed in triplicate (mean ± SD); the same below (**, *p* < 0.01; ***, *p* < 0.001); **(B)** The N protein expression level was detected using protein immunoblotting, *β-actin* was used as an internal reference, and the experiment was repeated at least twice.

To further verify the effect of miR-130c-5p on N protein expression in SHVV, we co-transfected CCO cells with analogs of miR-130c-5p, inhibitors and their controls and the constructed plasmid pCDNA3.1-N, respectively, and 24 h after transfection, we detected the mRNA expression of N proteins in the cells by qRT-PCR, and also measured the N protein expression levels by Western blot to determine the expression level of N protein. The results showed that overexpression of miR-130c-5p in CCO cells significantly decreased the N mRNA expression level, and conversely, inhibition of its expression up-regulated N mRNA expression (**Figure 4A**). In addition, overexpression of miR-130c-5p resulted in thinner protein bands of N protein detected by Western blot, and conversely, inhibition of its expression resulted in thicker protein bands (**Figure 4B**). These data suggest that miR-130c-5p can downregulate viral N expression, which will inhibit SHVV replication.

### 3.5 pCDNA3.1-N activates host innate immune responses

**Figure 5:**
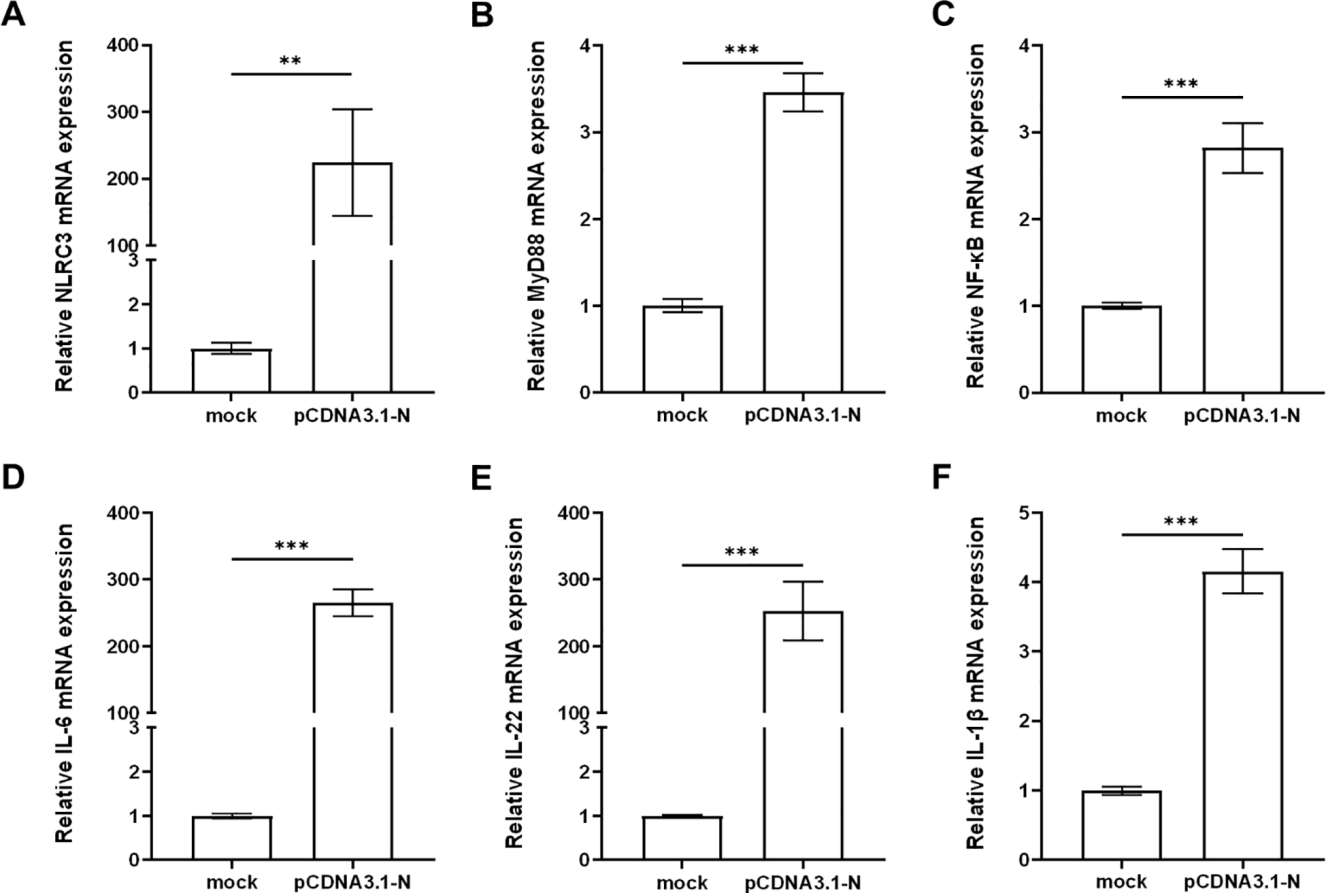
pCDNA3.1-N activated the host innate immune response. CCO cells were transfected with pCDNA3.1-N and harvested for 24 h after transfection. The mRNA expression levels of NLRC3 **(A)**, MyD88 **(B)**, NF-κB **(C)**, IL-6 **(D)**, IL-22 **(E)** and IL-1β **(F)** were measured using qRT-PCR. The *β-actin* was used as an internal control, and all the data represent the results of at least two independent experiments, and each assay was performed in triplicate (mean ± SD), the same as below (**, *p* < 0.01; ***, *p* < 0.001).

Studies have shown that nuclear factor kappa-B (NF-κB) plays an important role in the study of fish immune defense mechanisms as a key regulator targeting a variety of proteins and thus participating in the regulation of host responses (22, 23). To investigate the role of host immune response regulation in the SHVV infection, we constructed pCDNA3.1-N plasmid to mimic the expression of SHVV nuclear proteins and transfected it into CCO cells. Subsequently, the expression of immune genes related to the NF-κB related signaling pathway, including Nucleotide-oligomerization domain (NOD)-like receptor subfamily C3 (NLRC3), myeloid differentiation factor 88 (MyD88), NF-κB, and a variety of inflammatory cytokines (interleukin-6 (IL-6), interleukin-22 (IL-22), and interleukin-1beta (IL-1β)), was detected using qRT-PCR. The results showed that transfection of the pCDNA3.1-N plasmid significantly promoted the expression of these genes (**Figures 5A–F**). This indicated that the simulated expression of SHVV nuclear proteins activated the host’s antiviral immune response. The activation of this response is likely to be through the synergistic action of NF-κB mediated related signaling pathways to achieve host cell antiviral defense. In this study, it was known that miR-130c-5p can regulate the expression of nuclear proteins in SHVV, so whether miR-130c-5p is involved in these signaling pathways to affect viral infection and its specific regulatory role would require further investigation.

### 3.6 MiR-130c-5p regulates expression of immune factors associated with the NF-κB signaling pathway

**Figure 6:**
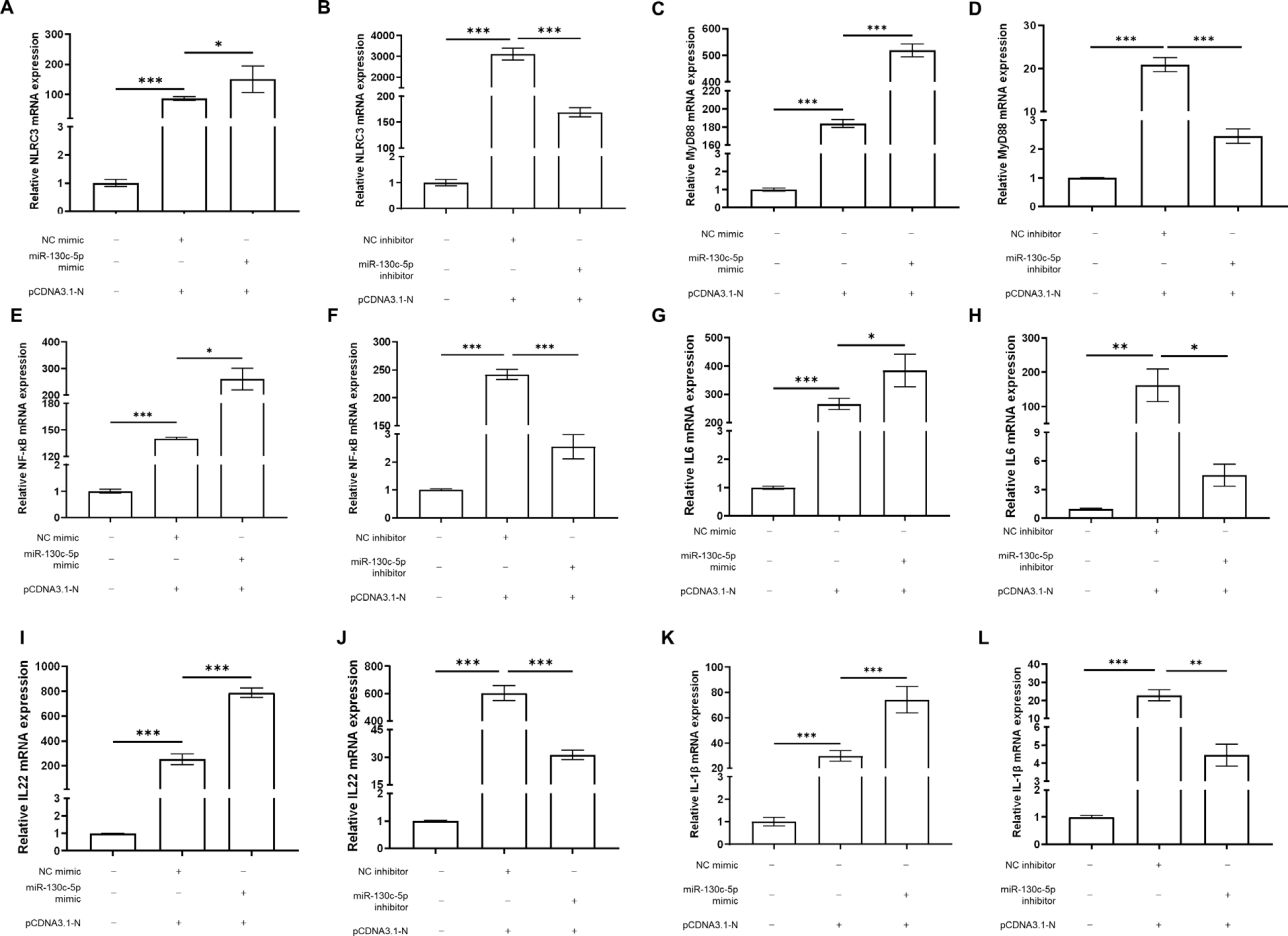
3.6MiR-130c-5p regulates expression of immune factors associated with the NF-κB signaling pathway. CCO cells were transfected with NC mimic, miR-130c-5p mimic, NC inhibitor, miR-130c-5p inhibitor, and pCDNA3.1-N, respectively. The cells were harvested 24 hours after transfection. The mRNA expression levels of NLRC3 **(A, B)**, MyD88 **(C, D)**, NF-κB **(E, F)**, IL-6 **(G, H)**, IL-22 **(I, J)** and IL-1β **(K, L)** were measured using qRT-PCR. The *β-actin* was used as an internal control, and all the data represent the results of at least two independent experiments, and each assay was performed in triplicate (mean ± SD), the same as below (*, *p* < 0.05; **, *p* < 0.01; ***, *p* < 0.001).

Given the established role of miR-130c-5p in regulating SHVV nuclear protein expression, we further investigated whether miR-130c-5p is involved in the NF-κB-mediated related signaling pathway affecting viral infection and its specific regulatory mechanisms. We transfected CCO cells with NC mimic, NC inhibitor, miR-130c-5p mimic or inhibitor and pCDNA3.1-N. Subsequently, the expression levels of NLRC3, MyD88, NF-κB, IL-6, IL-22 and IL-1β were detected. The experimental results showed that transfection of miR-130c-5p mimic significantly up-regulated the expression levels of NLRC3, MyD88, NF-κB, IL-6, IL-22, and IL-1β, which was more pronounced compared to transfection of pCDNA3.1-N alone (**Figures 6A–L**). In contrast, transfection of the miR-130c-5p inhibitor caused the opposite effect. These changes suggest that miR-130c-5p indirectly regulates relevant signaling pathways through these key regulators of immune response, further confirming the specificity of miR-130c-5p’s interaction with the *n* gene of SHVV and its role in regulating immune factor expression.

### 3.7 MiR-130c-5p upregulates the expression of immune-related genes in CCO cells

**Figure 7:**
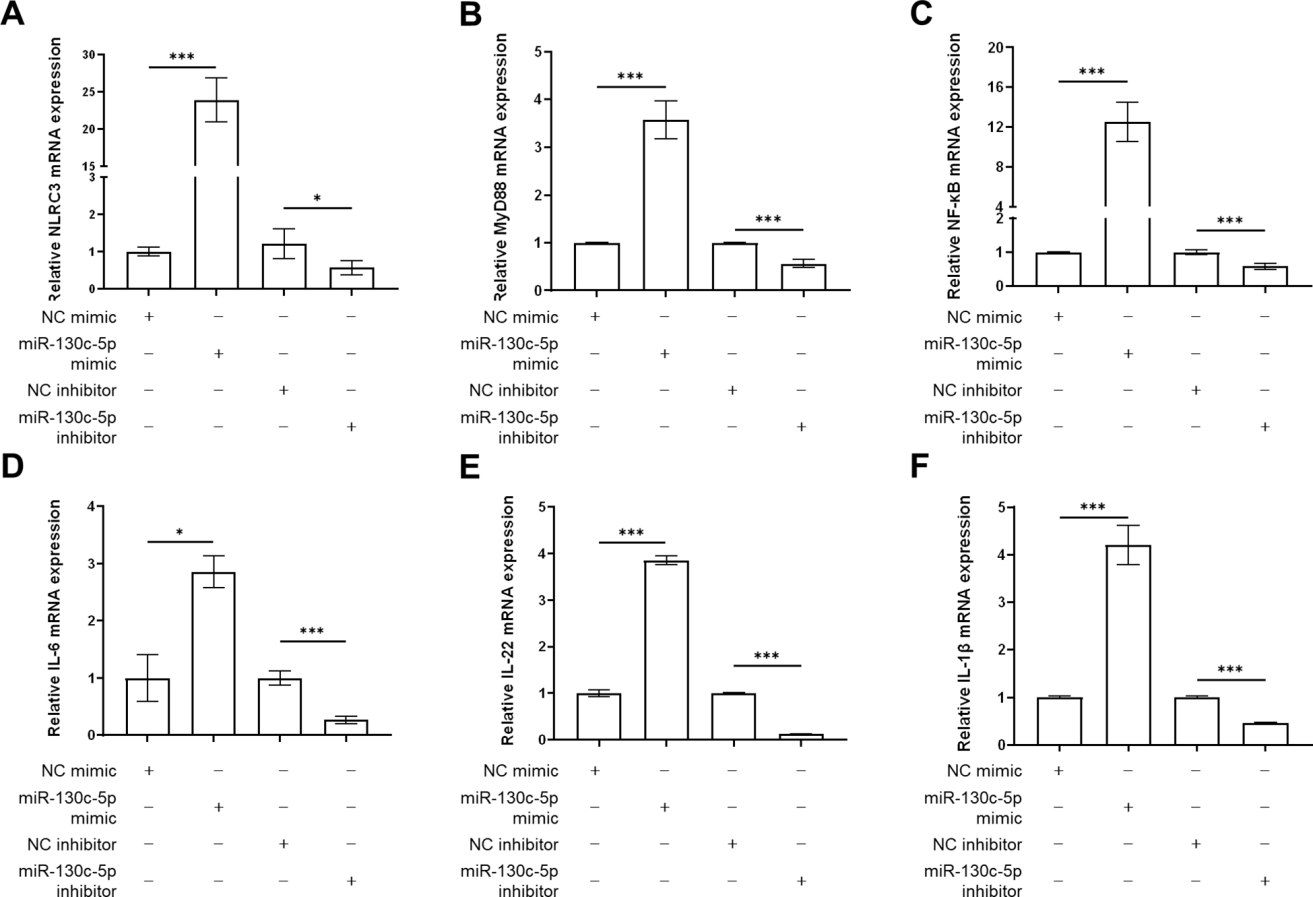
The effects of MiR-130c-5p on immune gene expression. CCO cells were transfected with NC mimic, miR-130c-5p mimic, NC inhibitor, and miR-130c-5p inhibitor, respectively, and the cells were harvested 24 hours after transfection. The mRNA expression levels of NLRC3 **(A)**, MyD88 **(B)**, NF-κB **(C)**, IL-6 **(D)**, IL-22 **(E)** and IL-1β **(F)** were measured using qRT-PCR. The *β-actin* was used as an internal control, and all the data represent the results of at least two independent experiments, and each assay was performed in triplicate (mean ± SD), the same as below (*, *p* < 0.05; ***, *p* < 0.001).

To further investigate the immune regulatory mechanism of miR-130c-5p on SHVV infection after viral infection of CCO cells, we transfected CCO cells with NC mimic, miR-130c-5p mimic, NC inhibitor, and miR-130c-5p inhibitor. Cells were harvested 24 h after transfection, and we used qRT-PCR to detect the expression levels of immune-related genes such as NLRC3, MyD88, NF-κB, IL-6, IL-22, and IL-1β. The results showed that overexpression of miR-130c-5p significantly promoted the expression of these immune genes, whereas inhibition of miR-130c-5p resulted in down-regulation of expression (**Figures 7A–F**). This indicated that miR-130c-5p may promote the transcription of NF-κB and various pro-inflammatory mediators through MyD88 and NLRC3 to participate in the relevant signaling pathways to respond to inhibit SHVV replication. In conclusion, overexpression of miR-130c-5p enhances the immune response of the host, which indirectly promotes the clearance of the virus; conversely, inhibition of miR-130c-5p results in a weakening of the host’s antiviral immune response.

## 4 Discussion

A number of studies have confirmed that microRNAs (miRNAs) are not only involved in various biological processes in eukaryotes, such as cell proliferation, development, differentiation and metabolism, and apoptosis, but also play an important regulatory role in the process of viral infection (24–27). Studies have shown that miRNAs are significantly altered by viral invasion, and this alteration of miRNAs is also closely related to the replication process of the virus and the immune response of the host (28, 29). For example, *Hepatitis C virus* (HCV) utilizes host cell miRNAs and regulates miRNA expression in infected hepatocytes in order to infect and multiply (30); *Classical swine fever virus* (CSFV) inhibits the expression of miR-140, which inhibits CSFV replication by binding to the host factor Rab25 (31); miR-34a and miR-361 inhibit foot-and-mouth disease virus (FMDV) replication by stimulating IFN-β promoter activity and activating the interferon-stimulated response element (ISRE), which in turn activates the PK-15 cellular immune response (32). In summary, viral infection alters host miRNA expression and, in turn, changes in miRNAs affect viral replication.

Earlier studies show that SHVV infection upregulates the expression of miR-130c-5p (21). However, the specific mechanism of miR-130c-5p’s role in SHVV infection remains poorly understood. In our study, we learned that miR-130c-5p was up-regulated during viral infection and overexpression of miR-130c-5p inhibited viral replication. This suggests that SHVV can replicate efficiently in CCO cells and that the production of miR-130c-5p by CCO may be an antiviral immune response. This result echoes findings in other studies that affect viral replication by regulating miRNA levels (33–35). Additionally, the results of dual luciferase assay showed that miR-130c-5p, as a novel antiviral factor, could effectively target and regulate the *n* gene to inhibit viral replication, and it could be a potential target for the development of novel therapeutic strategies against SHVV infection. To further verify whether miR-130c-5p plays a role in inhibiting SHVV replication, the experiments were performed by qRT-PCR and Western blot experiments, which concluded that miR-130c-5p indeed exerts an inhibitory effect on viral replication. Similar miRNA-targeted binding to viral genes has been reported. For example, miR-3145 targets the PB1 gene encoding polymerase basic protein 1 to inhibit influenza A virus replication (36); miR-181a-5p targets the stimulator of interferon genes (STING) to inhibit replication of Fowl adenovirus serotype 4 (FAdV-4) (37); miR-214 inhibits snakehead vesiculovirus replication by targeting the coding regions of viral N and P (3). The results that miR-130c-5p affects viral proliferation in host cells by targeting the viral genome are consistent with previous studies, and the ability of miRNAs to inhibit viral replication and infection by down-regulating the expression of key proteins has been demonstrated in several studies (38). In addition, studies have demonstrated that miRNAs exert their regulatory functions mainly through selective binding to complementary mRNAs, including direct inhibition of protein translation after partial binding of mature miRNAs to transcripts, leading to a decrease in transcription levels; and almost complete binding to transcripts, which affects the stability of viral mRNAs leading to protein degradation (39). The results of the present experiment are consistent with the latter, that SHVV shows down-regulation of expression at both the gene level and the protein level, and miR-130c-5p is degraded by affecting the stability of SHVV viral mRNA to the viral nucleoprotein. The innate immune response of the host is the first line of defense against microbial infections, and this process is mainly regulated by the NF-κB signaling pathway to activate and promote the expression of relevant immune genes. When viruses invade the host, immune cells rapidly produce key mediators such as interferon (IFN), chemokines, interleukins (IL), and tumor necrosis factor (TNF), thereby initiating antiviral innate immune and inflammatory responses.

Upon viral infection of the host, host immune cells produce mediators of antiviral innate immune and inflammatory responses such as interferon (IFN), chemokines, interleukins (IL) and tumor necrosis factor (TNF) (40, 41). In turn, the innate immune response, as the first line of host defense against different microorganisms, is mainly regulated by the NF-κB signaling pathway, which promotes the expression of target genes. Meanwhile, NF-κB is a downstream signaling molecule of the Toll-like receptor (TLR) signaling pathway, which plays an important role in the study of immune defense mechanisms in fish (42, 43). The results of this study revealed that miR-130c-5p and pCDNA3.1-N significantly up-regulated the expression of immune-related genes, such as NLRC3, MyD88, NF-κB, IL-6, IL-22, and IL-1β, in CCO cells. This indicates that miR-130c-5p may promote the expression of immune factors such as IL-6, IL-22, IL-1β, etc., and thus enhance the host immune response by indirectly regulating various signaling pathways such as TLR and NLR associated with these immune genes. Several studies have shown that fish NLRC3, a regulatory NLR, is involved in the regulation of mitogen-activated protein kinase (MAPK), NF-κB, autophagy, and IFN-I signaling pathways, and is involved in the regulation of inflammatory and pro-inflammatory cytokines during immune stimulation (44, 45). IL-6 and IL-22 are interleukins with a wide range of biological activities, they can promote the proliferation and differentiation of target cells, enhance anti-infective and cell-killing ability, regulate the expression of other cytokines and membrane surface molecules, and mediate immune response, inflammatory response (46, 47). In addition, miR-130c-5p mimic significantly inhibited SHVV replication infected viruses are recognized by the innate immune system, and TLRs on cells recognize pathogen-associated molecular patterns (PAMPs), which are activated by myeloid differentiation factor 88 (MyD88) to activate NF-κB production of IFN (48, 49). IFN is secreted extracellularly, binds to other cell surface receptors, and initiates cascade signaling via the Janus tyrosine kinase-signal converter and activator of transcription (JAK-STAT) pathway (50, 51). IL-6 and IL-22, as inflammatory factors, do not have antiviral activity per se, but can act on target cells through JAK-STAT signaling, which leads to the secretion of interferon-stimulated genes (ISGs) such as antimicrobial peptides (AMPs), serum amyloid A (SAA), beta-defensins (BDs), S100, etc. and contribute to the inflammatory response to defend against viral infections (52–54). Among them, IL-6 induces the differentiation of B cells and secretion of antibody proteins, and also induces the proliferation and differentiation of cytotoxic T lymphocytes, and together with TNF-α and IL-1β constitutes the first defense barrier of the body’s immune response (55, 56). This demonstrated that miR-130c-5p in this study was not only involved in the increased expression of genes involved in the immune response, but also in the regulation of multiple signaling pathways, such as NF-κB, MyD88, TLR, NLR, and JAK-STAT, to induce enhancement of the viral immune response in the host. Although this study clearly demonstrated that miRNAs can not only influence the strength and effect of immune response by regulating the expression of immune factors, but also inhibit viral replication by directly targeting the genome of viral major structural proteins. However, in order to fully analyze the role of miR-130c-5p in the regulatory network, it is necessary to deeply explore the mechanism of its interactions with immune genes and clarify its direct targets.

**Figure 8:**
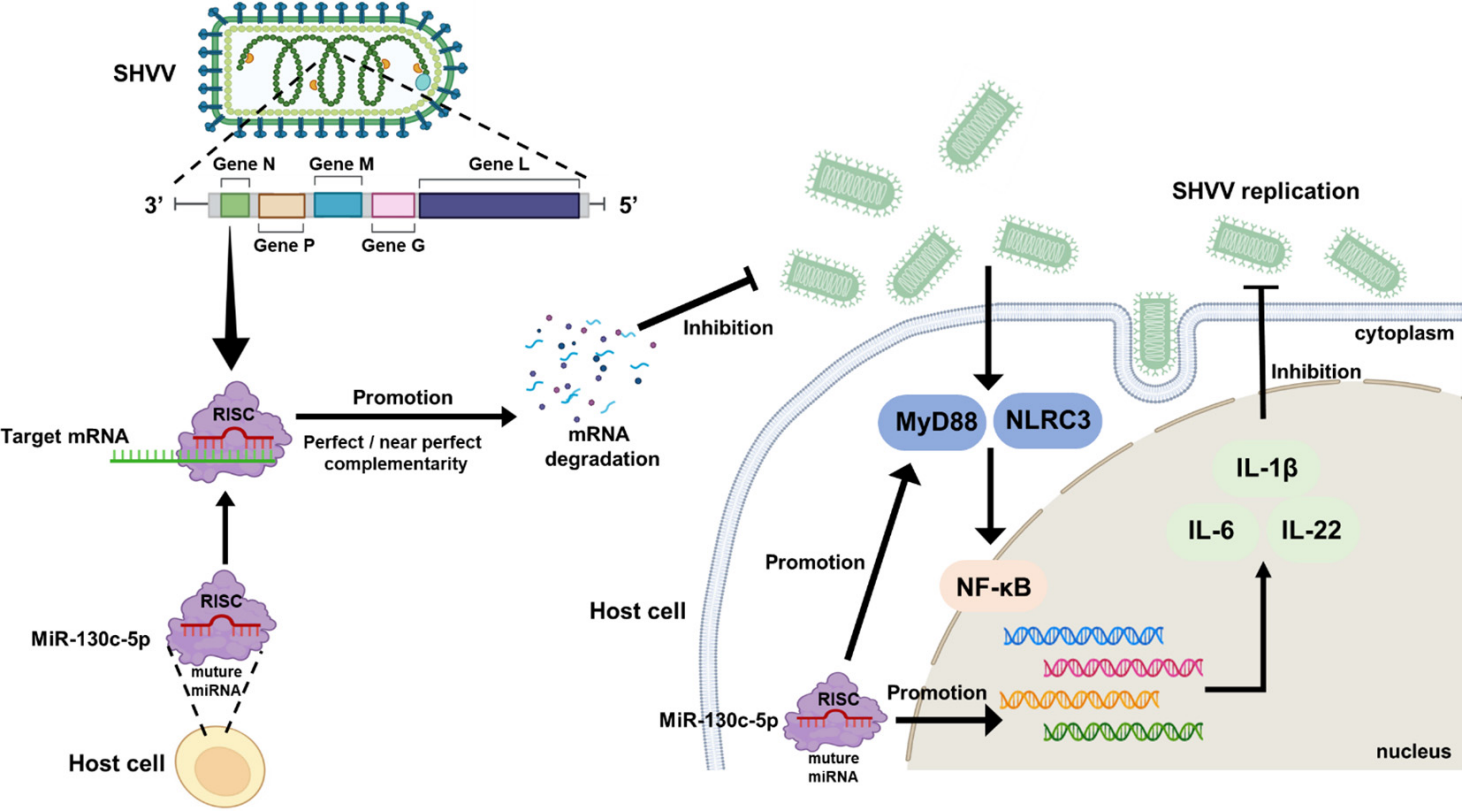
Antiviral mechanism of miR-130c-5p for SHVV infection. When SHVV invades cells, miR-130c-5p directly targets the viral n-gene to inhibit its replication; miR-130c-5p can activate TLRs and NLRs signaling pathways, mainly by inducing the expression of MyD88 and NLRC3, activating the NF-κB signaling pathway, up-regulating the key immune factors, such as IL-6, IL-22, and IL-1β, and significantly enhancing the host antiviral immune response; SHVV infection can activate the above signaling pathways to participate in the host antiviral immune response.

In summary, miR-130c-5p directly targets the viral *n* gene to inhibit SHVV replication during SHVV infection of CCO cells. In addition, overexpression of miR-130c-5p would up-regulate the expression of immune-related genes (IL-6, IL-22, and IL-1β) and interfere with viral replication by participating in the regulation of multiple signaling pathways, including NF-κB, MyD88, TLR, NLR, and JAK-STAT (**Figure 8**). Combining the findings of previous studies with those of the present study, the present study reveals the critical role of miR-130c-5p in virus-host interactions, providing new insights into the mechanism of SHVV infection and searching for potential targets for anti-SHVV drugs.

## Data Availability

The original contributions presented in the study are included in the article/supplementary material, further inquiries can be directed to the corresponding author(s).
